# Powassan Meningoencephalitis: A Case Report Highlighting Diagnosis and Management

**DOI:** 10.7759/cureus.16592

**Published:** 2021-07-23

**Authors:** Jolanta J Pach, Adeel S Zubair, Christopher Traner, Guido J Falcone, Jeffrey J Dewey

**Affiliations:** 1 Neurology, Yale School of Medicine, New Haven, USA

**Keywords:** powassan virus, viral meningitis, viral encephalitis, chronic lymphocytic leukemia, tick-borne flavivirus

## Abstract

Powassan virus (POWV), a rare flavivirus that may be transmitted by a tick bite, causes rare but severe cases of encephalitis, meningitis, and meningoencephalitis in humans. We present the case of a 62-year-old man with prior Lyme disease and reactive arthritis who presented to the hospital with symptoms of fever, headache, and fatigue. The patient developed rapid deterioration of mental status including profound expressive aphasia and required intubation and high-dose steroids. Cerebrospinal fluid (CSF) serologies were found to be positive for the POWV.

## Introduction

Infections of the Powassan virus (POWV) have been described in the Northern United States, Canada, and Russia [1]. The virus is transmitted by ixodid ticks and causes severe encephalitis and meningitis in humans. While rare, infections with POWV carry a 10-15% fatality rate, with some reports exceeding 20% [2]. Long-term sequelae of disease are also common [3, 4]. The symptoms reported most frequently in POWV case studies include fever, headache, confusion, generalized weakness, encephalopathy, neurological symptoms, focal deficit, and vomiting [5]. Common long-term sequelae include generalized weakness, cognitive difficulties, speech difficulties, imbalance and difficulty walking, spastic quadriplegia, ophthalmoplegia, and headaches [6]. Fewer than 300 cases of POWV have been reported since the virus was first discovered in 1958, but cases have been steadily increasing in the United States over the past decade [2, 3]. Serologic testing is the gold standard for diagnosis, specifically immunoglobulin M (IgM) antibody testing utilizing enzyme-linked immunosorbent assay and immunofluorescence antibody. However, only state health departments and the CDC can perform Powassan-specific serologic testing [5]. There is currently no treatment or vaccine available, and supportive care is the mainstay of treatment. We present a case of a 62-year-old man presenting with POWV meningoencephalitis and discuss the workup of such patients, as well as the progression of imaging findings often reported with POWV, characterized by the appearance and subsequent resolution of radiologic hyperintensity on T2-weighted MRI. Little is known about conditions that might predispose patients to severe POWV and its long-term consequences. Given the patient’s past medical history of chronic lymphocytic leukemia (CLL), we discuss the role of immunosuppression in neuroinvasive infection and advocate for a higher index of suspicion for neuroinvasive infection in immunosuppressed patients.

## Case presentation

A 62-year-old man with a past medical history significant for CLL, Lyme disease, and reactive arthritis presented to the hospital with fevers, headaches, and fatigue. At baseline, the patient was highly functioning and worked as a financial manager. He was not on any medications for his CLL. The patient's wife reported that the previous year he was diagnosed with reactive arthritis and started on methotrexate, which he discontinued 10 days prior to presentation on the advice of a holistic care provider in favor of a probiotic. In late May, he was otherwise in his normal state of health until a few days prior to presentation when he developed fatigue and night sweats. Subsequently, he developed headaches, malaise, and fever, at which point he presented to our hospital. His temperature was 102.1 Fahrenheit, pulse 94 beats per minute, blood pressure 158/84 mmHg, and oxygen saturation 95% on room air. Urgent cerebrospinal fluid (CSF) analysis showed 122 RBCs per microliter, 59 white blood cells per microliter (88% lymphocytes, 3% polymorphonuclear cells, 9% monocytes), protein of 98.9 mg/dL, and a glucose of 80 mg/dL (Table 1). CSF and blood cultures were obtained. A broad panel of additional CSF and serum labs was ordered as detailed in Table 2.

**Table 1:** Cerebrospinal fluid profile. CSF profile on day of presentation, suggestive of viral meningitis. CSF: Cerebrospinal fluid; PMNs: Polymorphonuclear cells.

CSF	Tube 1	Tube 4	Reference range
Appearance	No xanthochromia	No xanthochromia	No xanthochromia
RBCs	122	9	None cells/uL
WBCs	59	50	<6 cells/uL
Differential (%)	88% lymphocytes, 3% PMNs, 9% monocytes	74% lymphocytes, 24% monocytes, 1% PMNs, 1% basophils	
Protein	98.9		15-45 mg/dL
Glucose	80		40-70 mg/dL

**Table 2:** Serum and cerebrospinal fluid tests. Serum and CSF tests were negative for many potential causes of meningoencephalitis but serum IgM eventually returned positive for Powassan virus. The SS-A serology test was likely either a false positive or consistent with the patient’s history of reactive arthritis. Ab: Antibody; Ag: Antigen, PCR: Polymerase chain reaction; IgG: Immunoglobulin G; ANA: Anti-nuclear antibody; IFA: Indirect fluorescent antibody; ANCA: Antineutrophil cytoplasmic antibodies; dsDNA: Double-stranded DNA; IgM: Immunoglobulin M.

Test	Reference range	Result
Lactate dehydrogenase (serum)	122-241 U/L	237
C-reactive protein (serum)	0.0-1.0 mg/dL	0.4
HIV 1,2 Ab (serum)	Negative	Negative
HIV p24 Ag (serum)	Not detected	Not detected
Lyme Ab with Western Blot reflex (serum)	<0.90 LI	0.18
Anaplasma PCR (serum)	Negative	Negative
Babesia PCR (serum)	Negative	Negative
Babesia smear (serum)	Negative	Negative
Enterovirus PCR (CSF)	Not detected	Not detected
Adenovirus PCR (serum)	Not detected	Not detected
Direct fluorescent Ab PCR including influenza A and B, metapneumovirus, rhinovirus, parainfluenza virus, respiratory syncytial virus, Chlamydia pneumoniae, and Mycoplasma (nasal swab)	Negative	Negative
Influenza H1N1 (2009) PCR (serum)	Negative	Negative
West Nile IgG (CSF)	Negative	Negative
Herpes simplex virus 1,2 PCR (CSF)	Not detected	Not detected
CSF culture	Negative	Negative
Cryptococcal Ag (CSF)	Not detected	Not detected
Cytomegalovirus PCR (CSF)	Not detected	Not detected
Leptospira Ab (serum)	Negative	Negative
Hepatitis general panel (Hepatitis B surface Ag, Ab, C Ab with PCR reflex, A Ab) (serum)	Negative	Negative
ANA by IFA with reflex (serum)	<1:80	<1:80
ANCA screen with reflex (serum)	Negative	Negative
dsDNA with reflex (serum)	<10 IU/mL	1.7
QuantiFERON-TB (serum)	Negative	Negative
Quantitative buffy coat for blood parasites screen (serum)	Negative for intra-erythrocyte parasites	Negative for intra-erythrocyte parasites
Serotonin (serum)	56-244 ng/mL	25
Rheumatoid factor (serum)	<14 IU/mL	<10
SS-A (serum)	<7.0 U/mL	2.6
SS-B (serum)	<7.0 U/mL	<0.3
Autoimmune encephalopathy panel (serum and CSF)	Negative	Negative
St. Louis encephalitis IgM (CSF)	Negative	Negative
Eastern equine encephalitis IgM (CSF)	Negative	Negative
Western equine encephalitis IgM (CSF)	Negative	Negative
Powassan IgM sent to CDC (serum)	Negative	>320

**Figure 1:**
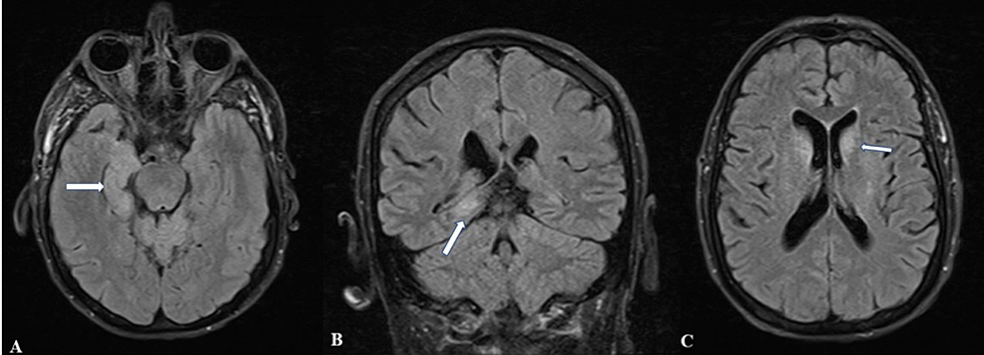
Initial MRI of the brain. MRI of the brain showing FLAIR hyperintensities in the right temporal lobe (panel A and B) as well as in the bilateral caudate nucleus (panel C). FLAIR: Fluid-attenuated inversion recovery.

**Figure 2:**
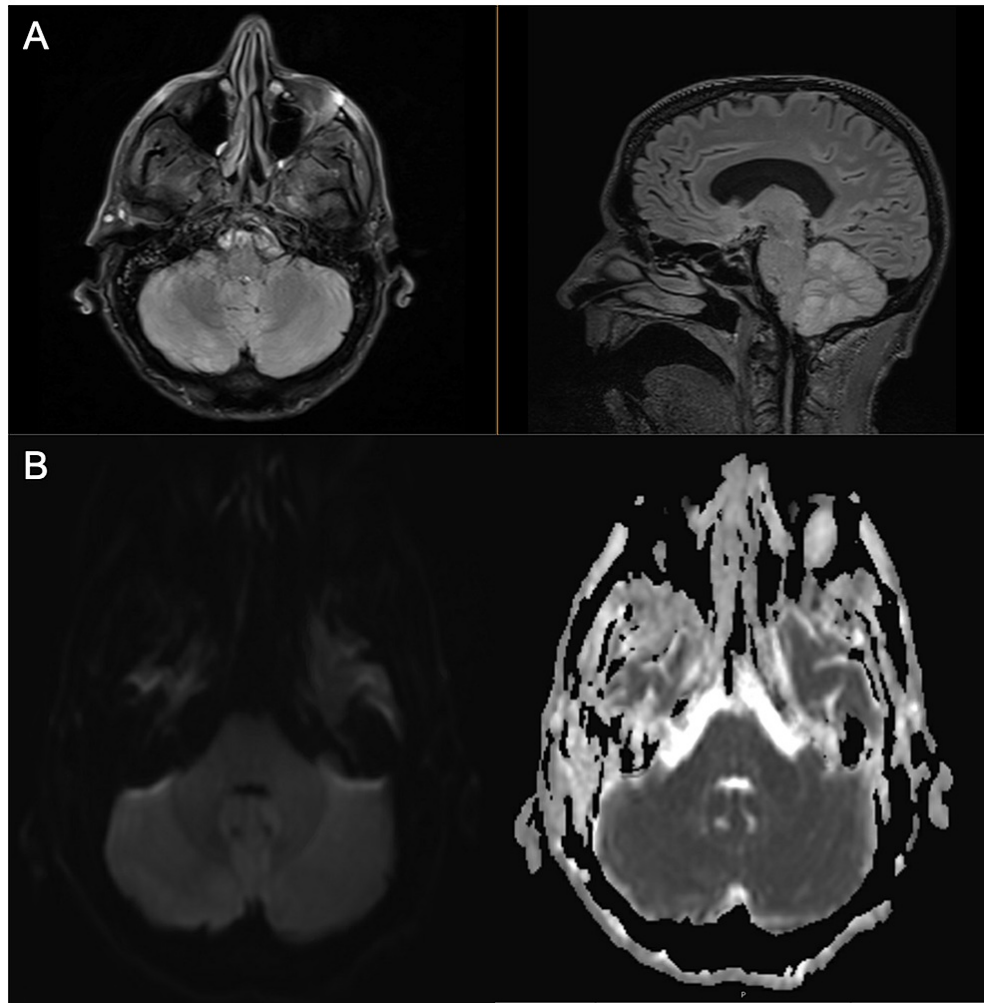
Follow-up MRI of the brain. A) MRI of the brain showing evidence of cerebellar FLAIR enhancement, consistent with cerebellitis B) DWI image with corresponding ADC map showing mild restricted diffusion. ADC: Apparent diffusion coefficient; DWI: Diffusion-weighted imaging; FLAIR: Fluid-attenuated inversion recovery.

Empiric antibiotics (vancomycin and ceftriaxone) and antiviral (acyclovir) were started for possible meningitis or encephalitis. CT scans of the chest, abdomen, and pelvis were performed due to a history of CLL, which showed no evidence of bulky lymphadenopathy. A peripheral blood smear was consistent with chronic CLL. MRI of the brain with and without contrast showed mild generalized cerebral and cerebellar atrophy and a few small nonspecific T2 fluid-attenuated inversion recovery (FLAIR) hyperintensities with no enhancement on T1-weighted MRI (Figure 1). An overnight continuous EEG did not reveal any seizures. The patient’s mental status deteriorated over the hospital day 1-2 and he developed profound expressive aphasia and ataxia. He required a non-rebreather to maintain oxygen saturation above 90% because of failure to protect his airway. He was transferred to the ICU and intubated for airway protection on hospital day 6. Given that his CSF bacterial cultures showed no growth to this point, and herpes simplex virus (HSV) and varicella-zoster virus (VZV) polymerase chain reactions (PCRs) were negative, empiric antibiotics and acyclovir were discontinued and he was given a five-day course of IV methylprednisolone with gradual improvement in neurologic status. MRI brain with gadolinium was repeated and showed extensive high signal with mild restricted diffusion involving bilateral cerebellar hemispheres, consistent with cerebellitis (Figure 2).

Subsequently, CSF serologies returned positive for POWV, which supported a diagnosis of Powassan meningoencephalitis. The infectious diseases service was consulted and recommended no further treatment in the in-patient setting. After hospital day 34, the patient was transferred to a skilled nursing facility and underwent physical and occupational therapy.

**Figure 3:**
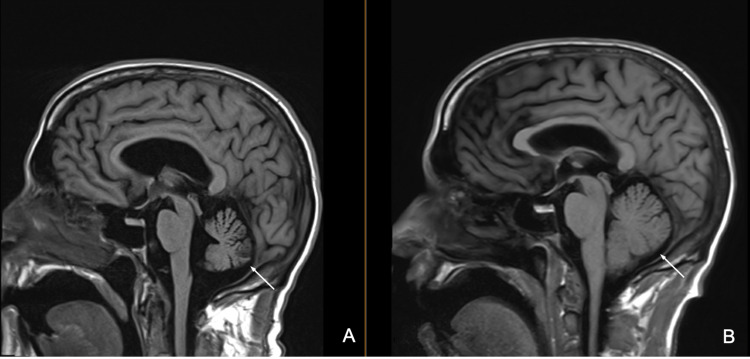
One-year follow-up MRI of the brain. Follow-up MRI one year after initial presentation showing progressive cerebellar atrophy (A) compared to previous imaging (B) with an improvement of hyperintensities.

Upon evaluation in the out-patient clinic a few weeks later, the patient demonstrated significant left-sided ataxia and expressive aphasia. On subsequent three-month follow-up, he had developed spasticity of both upper extremities, resulting in difficulty using a keyboard and occasional pain in the left upper extremity. On this examination, he was noted to have increased tone of the left upper extremity and flexed posture of biceps and fingers. Speech therapy was recommended and physical and occupational therapy was continued. He was prescribed gabapentin 300 mg three times daily (TID) and baclofen 10 mg TID, and referred for botulinum toxin (Botox) injections. A year after his initial admission, the patient underwent a follow-up MRI which showed progressive cerebellar atrophy compared to previous imaging with full resolution of hyperintensities (Figure 3).

Tone improved in both upper extremities with baclofen and Botox injections, and his wife noted he was not complaining of pain on this regimen. However, the patient continued to have flexed posture of the 4th and 5th digits of both hands and developed a head drop with decreased neck extensor tone. Botox regimen was adjusted to include these muscle groups with partial effect. To date, the patient has continued with Botox injections every three months and underwent a procedure to receive a baclofen pump for refractory bilateral upper extremity spasticity with subsequent improvement in his stiffness and tone. He is wheelchair-bound and continues to have ataxia and expressive aphasia but is alert and oriented and able to follow commands.

## Discussion

The extent of neurologic involvement seen in POWV infection varies widely. Neurologic symptoms seen in POWV include altered mental status, seizures, headache, memory impairment, blurry vision, diplopia, nystagmus, upward gaze palsy, dizziness, spastic and flaccid paralysis, and positive Babinski sign [5, 7]. Upper and lower motor neuron involvement is more significant in POWV infection than in other causes of viral encephalitis [5]. Additionally, there is some evidence that POWV may be similar to hemorrhagic viruses such as dengue and yellow fever, as some cases of POWV show intraparenchymal hemorrhage and subdural hematoma [5]. Thus, POWV may also present with focal deficits such as hemiplegia and hemiparesis in the setting of intracranial bleeding. Neuropsychiatric symptoms such as anhedonia and depression may also be seen. Long-term sequelae are variable, ranging from severe, disabling residual deficits to a return to near-baseline function with a normal neurologic exam [8]. As noted earlier, our patient developed expressive aphasia and spasticity of both upper extremities for which he underwent physical, occupational, and speech therapy as well as treatment with an oral muscle relaxant and Botox injections. Apart from neurologic symptoms, respiratory failure may also occur from depressed consciousness leading to failure to protect the airway or inadequate oxygenation. Intubation may be needed, as demonstrated by our case [9].

Radiologic findings in POWV meningoencephalitis also vary widely. In the acute setting, a CT scan is unlikely to show abnormalities unless there is intracranial bleeding [5]. MRI is more likely to show ischemia on T2 FLAIR but these are not universal. Hyperintensity is most often seen in the periventricular white matter, perivascular space, and deep white matter. These abnormalities tend to improve once symptoms have resolved [5, 8]. Abnormal contrast enhancement is not typically seen, though subtle enhancement of the vermis and diffuse cerebellar parenchymal and leptomeningeal enhancement have been reported in some cases [8]. The progression of MRI findings over time with simultaneous gradual improvement in our patient’s neurologic status agrees with imaging findings of POWV neuroinvasive disease reported in the literature. T2 FLAIR hyperintensities were seen and subsequently resolved on repeat imaging concurrently with improvements in the patient’s neurologic status. While initial imaging revealed hyperintensities involving the bilateral hippocampal/parahippocampal gyrus and caudate heads, subsequent scans showed resolution of these findings with a new extensive high signal with mild restricted diffusion involving bilateral cerebellar hemispheres.

Our patient’s history of CLL is an interesting confounder in this case. Immunosuppression secondary to CLL leads to an increased risk of infections, even for patients not undergoing immunosuppressive treatment. This may be in part due to the impaired T-cell immune response and hypogammaglobulinemia known to accompany CLL [10]. Thus, patients with CLL, even those like our patient who is not on immunosuppressive medications, are prone to new infections or reactivations of viral infections. This underscores the importance of a higher index of suspicion for neuroinvasive infection in all immunosuppressed patients.

Although infection with POWV is rare, the potential for rapid clinical deterioration and the need for urgent supportive care warrant consideration of POWV when tick-borne illness is suspected, particularly in the Northeastern United States and Great Lakes regions. As demonstrated by our case, POWV can cause severe meningitis and encephalitis with serious long-term neurologic sequelae. Though our patient survived, POWV infection can be fatal in up to 20% of cases, underscoring the importance of early supportive care. Physicians should be prepared to recognize the common presenting symptoms of POWV, including fever, headache, confusion, encephalopathy, and neurological symptoms, and to provide both aggressive supportive care in the initial phases and appropriate management of long-term neurologic sequelae. In many cases, multidisciplinary care with occupational, physical, and/or speech therapy may be helpful. Patient education is also important, as no vaccination and specific treatment exist at this time, and tick bite prevention remains the best defense against POWV infection.

## Conclusions

It is important to have a broad differential diagnosis when evaluating patients presenting with symptoms of neuroinvasive infection. While rare, as demonstrated by our patient’s case, infection with POWV may lead to rapid clinical deterioration and long-term neurologic deficits. Our patient exhibited ataxia, expressive aphasia, and spasticity as consequences of severe POWV infection. Immunosuppression may play a role in a patient’s susceptibility to neuroinvasion with POWV. A thorough history, clinical examination, and diagnostic testing are necessary to guide early supportive care. Appropriate outpatient rehabilitation and management of sequelae can minimize long-term morbidity. Patient education remains the best preventive strategy.
